# Asymmetric dimethylarginine mediates oxidative stress and atrial remodeling in HL-1 cells

**DOI:** 10.3389/fmed.2025.1696845

**Published:** 2025-11-04

**Authors:** Chengyun Yu, Ming Zhang, Wei Xia

**Affiliations:** ^1^Department of Cardiology, Songjiang Hospital Affiliated to Shanghai Jiao Tong University School of Medicine, Shanghai, China; ^2^School of Clinical Medicine, Shandong Second Medical University, Weifang, China; ^3^Department of Cardiology, Qingdao Municipal Hospital, University of Health and Rehabilitation Sciences, Qingdao, China

**Keywords:** asymmetric dimethylarginine, oxidative stress, TGF-β1, atrial remodeling, atrial fibrillation

## Abstract

**Introduction:**

Atrial fibrillation (AF) is a common cardiac arrhythmia, and endothelial dysfunction and oxidative stress (OS) are key mechanisms promoting atrial remodeling. Asymmetric dimethylarginine (ADMA) inhibits nitric oxide synthase (NOS) but its role in AF-related atrial remodeling remains unclear.

**Methods:**

Mouse atrial myocyte HL-1 cells were treated with ADMA, H_2_O_2_, N-acetylcysteine (NAC), or their combinations. Cell viability, reactive oxygen species (ROS) levels, and TGF-β1 expression were detected using CCK-8, flow cytometry, fluorescence microscopy, and Western blot. A clinical cohort study included 60 AF patients and 30 controls to measure serum ADMA, TGF-β1, and NO levels.

**Results:**

ADMA (30 μM) significantly increased ROS generation and upregulated p47phox and TGF-β1 expression in HL-1 cells, which was reversed by NAC. AF patients had higher serum ADMA and TGF-β1 levels and lower NO levels than controls (*P*<0.01).

**Discussion:**

ADMA may induce TGF-β1 expression by enhancing NOX-ROS levels, leading to myocardial oxidative damage and atrial remodeling, which provides new insights into AF pathophysiology.

## 1 Introduction

Atrial fibrillation (AF) is the most prevalent sustained cardiac arrhythmia of clinical significance, with its incidence rising globally, partly due to an aging population (1). The pathological mechanisms underlying AF primarily involve the interplay of multiple factors, including electrophysiological abnormalities and structural remodeling, which encompasses fibrosis and atrial enlargement. The mechanisms associated with atrial fibrosis primarily include various signaling pathways, such as oxidative stress (OS), the renin-angiotensin system (RAS), transforming growth factor-β1 (TGF-β1), inflammation, and matrix metalloproteinases (MMPs) (2). Furthermore, it is increasingly recognized that cardiovascular physiological disturbances, particularly endothelial dysfunction, significantly contribute to atrial remodeling (3, 4).

Endothelial dysfunction is clinically characterized by reduced nitric oxide (NO) bioavailability, a well-documented feature in patients with AF (5, 6). Asymmetric dimethylarginine (ADMA), an endogenous nitric oxide synthase (eNOS) inhibitor, competes with L-arginine for NOS binding, thereby suppressing NO production and impairing endothelial function (7, 8). Evidence suggests that ADMA exacerbates oxidative stress via the Sirt1/Foxo3 pathway, as observed in diabetic retinopathy (9), and activates ROCK in endothelial cells, compromising cell viability and promoting vascular remodeling (10). We hypothesize that in atrial tissue, ADMA-driven endothelial dysfunction and oxidative stress synergistically enhance structural vulnerability and remodeling, thereby facilitating the initiation and perpetuation of AF.

This study demonstrated that ADMA significantly elevated reactive oxygen species (ROS) levels in HL-1 mouse atrial myocytes, accompanied by upregulated p47phox and TGF-β1 protein expression, which are markers of endothelial dysfunction. Notably, these effects were reversed by the ROS scavenger N-acetylcysteine (NAC). Consistent with these *in vitro* findings, our clinical cohort study (60 AF patients, 30 controls) showed elevated ADMA and TGF-β1 along with reduced NO in AF patients (*P* < 0.01). Our integrated findings suggest that ADMA promotes atrial remodeling through multiple molecular pathways, thereby increasing susceptibility to AF. This mechanistic network provides novel insights into the pathophysiology of AF and identifies potential therapeutic targets.

## 2 Materials and methods

### 2.1 Cell culture

The HL-1 mouse atrial myocytes (CC9097, Cellcook Biotech, China) were cultured in DMEM (11965092, Gibco, China) supplemented with penicillin (100 U/mL, CM1005-005, Cellcook, China), streptomycin (CM1005-005, 100 μg/mL, Cellcook, China), and 10% fetal bovine serum (FBS, Gibco, United States, 10100147) at 37 °C in a 5% CO_2_ atmosphere. Certain experimental groups were treated with serum-free medium (high glucose basal medium only) for 4–8 h. Subsequently, the cells were randomly assigned to different groups for drug intervention, which lasted for 24 h prior to the relevant experiments. ADMA, H_2_O_2_ and NAC conditions for *in vitro* studies were established as confirmed by the CCK8 assay.

### 2.2 Cell proliferation activity detection

After trypsinization, HL-1 cells were resuspended in serum-free DMEM and seeded in 96-well plates at a density of 4 × 10^4^ cells/mL (100 μL per well). When the cell confluence reached 60%–70%, the medium was replaced with solutions containing ADMA at concentrations of 3, 10, 30, 50, or 100 μM or with control medium devoid of ADMA. Following a 24-h incubation, the cells were washed with PBS and incubated with 10 μL of CCK-8 for 1 h. The optical density (OD) at 450 nm was then measured. Cell viability (%) was calculated using the following formula: Viability = [(OD_exp−OD_blk)/(OD_ctrl−OD_blk)] × 100%.

### 2.3 ROS detection

HL-1 cells were seeded in 6-well plates and cultured to 60%–70% confluence. To investigate the effect of ADMA on OS, the cells underwent a 24-h treatment with varying concentrations of ADMA (0, 3, 10, 30, 50, and 100 μM). Following this incubation, the production of intracellular ROS was evaluated using the fluorescent probe 2’,7’-dichlorodihydrofluorescein diacetate (DCFH-DA). Specifically, the culture medium was replaced with a serum-free medium containing 10 μM DCFH-DA, and the cells were incubated at 37 °C for 30 min in the dark. After incubation, the cells were gently washed twice with PBS to eliminate any excess probe. ROS levels were quantified using two complementary methods. First, fluorescence microscopy was employed for qualitative and semi-quantitative analysis. For robust quantitative analysis, flow cytometry was performed. The cells were incubated with DCFH-DA for 30 min at 37 °C, washed with PBS, and analyzed using fluorescence microscopy, capturing representative images per well with ImageJ. Additionally, flow cytometry was performed with an excitation wavelength of 495 nm and emission at 525 nm (FL1 channel), analyzed using FlowJo 10.9.0.

### 2.4 Western blots

For the separation of proteins from HL-1 cells, we employed RIPA buffer supplemented with a protease inhibitor mixture and a phosphatase inhibitor mixture at a ratio of 1:50. The resulting mixture was incubated on ice for 10 min, followed by centrifugation at 12,000 *g* for 15 min. Protein concentration was quantified using the BCA kit (Beyotime, P0011). Subsequently, proteins were transferred to PVDF membranes (ISEQ00005, Millipore) in accordance with the adiabatic SDS-PAGE protocol (10%) and incubated with the specified primary antibodies at 4 °C. After incubation, membranes were washed three times with TBST. The membranes were then treated with HRP-conjugated secondary antibodies for 1 h. Protein detection was performed using the Immobilon^®^ UltraPlus Western HRP Substrate (WBULP, Millipore) in conjunction with an automated chemiluminescent imaging system. Band quantification was conducted utilizing ImageJ software. The antibodies used in the Western blot analysis included: rabbit monoclonal anti-TGFβ1 (1:1000, ab215715, Abcam), rabbit monoclonal anti-p47phox (1:1000, ab308256, Abcam), rabbit monoclonal anti-GAPDH (1:10000, ab181602), goat anti-rabbit IgG (1:20000, 111-035-003, Jackson), and goat anti-mouse IgG (1:20000, 115-035-003, Jackson).

### 2.5 Baseline characteristics of the AF patients and control

Baseline Characteristics, Medication and Laboratory examination were gathered. Baseline Characteristics: age, gender, Hypertension, Diabetes, History of old MI, Old Stroke. Medication: AECI or ARB, Betablocker, Statins. Laboratory examination: Creatinine, TG, LDL-C.

### 2.6 Biochemical assay

Enzyme-linked immunosorbent assay (ELISA) kits or metabolism assay kit were used to determine the levels of serum ADMA, TGF-β1 and NO following the manufacturer’s instructions (Elabscience, China, Catalog Number E-EL-0042, E-EL-0162, E-BC-K035-M, respectively).

### 2.7 Statistical analysis

The Shapiro-Wilk test and *F*-test were applied to all data prior to conducting parametric tests. Continuous data are presented as mean ± standard deviation (SD). After assessing normality and homogeneity of variance, continuous data were compared using unpaired Student’s *t*-test or one-way analysis of variance (ANOVA). *Post hoc* analyses following one-way ANOVA were conducted using Tukey’s post-hoc test. Quantification was performed using ImageJ software, while GraphPad Prism 9 and Photoshop 2021 were utilized for graphing.

## 3 Results

### 3.1 ADMA promotes atrial remodeling in HL-1 cells

**FIGURE 1:**
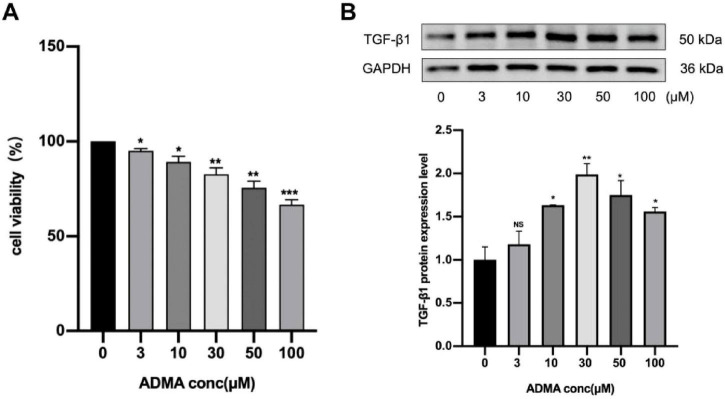
Asymmetric dimethylarginine (ADMA) promotes atrial remodeling in HL-1 cells. **(A)** The viability of HL-1 cells was evaluated under varying ADMA concentrations (0, 3, 10, 30, 50, 100 μM). **(B)** Representative images and a comparison of transforming growth factor-β1 (TGF-β1) protein levels from western blots in six groups. *n* = 3 per group. Data are presented as mean ± SD. Statistical significance was determined using One-way ANOVA, and Tukey’s post-hoc test. *P < 0.05, **P < 0.01 vs. the normol control group.

To investigate atrial remodeling, we evaluated the levels of TGF-β1 protein associated with atrial remodeling in the HL-1 cell model. Firstly, HL-1 cells were subjected to serum starvation and subsequently exposed to varying concentrations of ADMA (0, 3, 10, 30, 50, and 100 μM) for a duration of 24 h. The results from CCK-8 assays indicated a dose-dependent decrease in cell viability across all ADMA treatment groups (*P* < 0.05), with the group treated with 100 μM ADMA exhibiting the lowest survival rate (Figure 1A). TGF-β1 expression was significantly upregulated compared to the control group (*P* < 0.01) when treated with ADMA at a concentration of 30 μM. In contrast, treatment with ADMA at 100 μM resulted in a marked reduction in TGF-β1 expression (*P* < 0.01) (Figure 1B). These findings suggest that ADMA promotes atrial remodeling in HL-1 cells.

### 3.2 ADMA promotes oxidative stress in HL-1 cells

**FIGURE 2:**
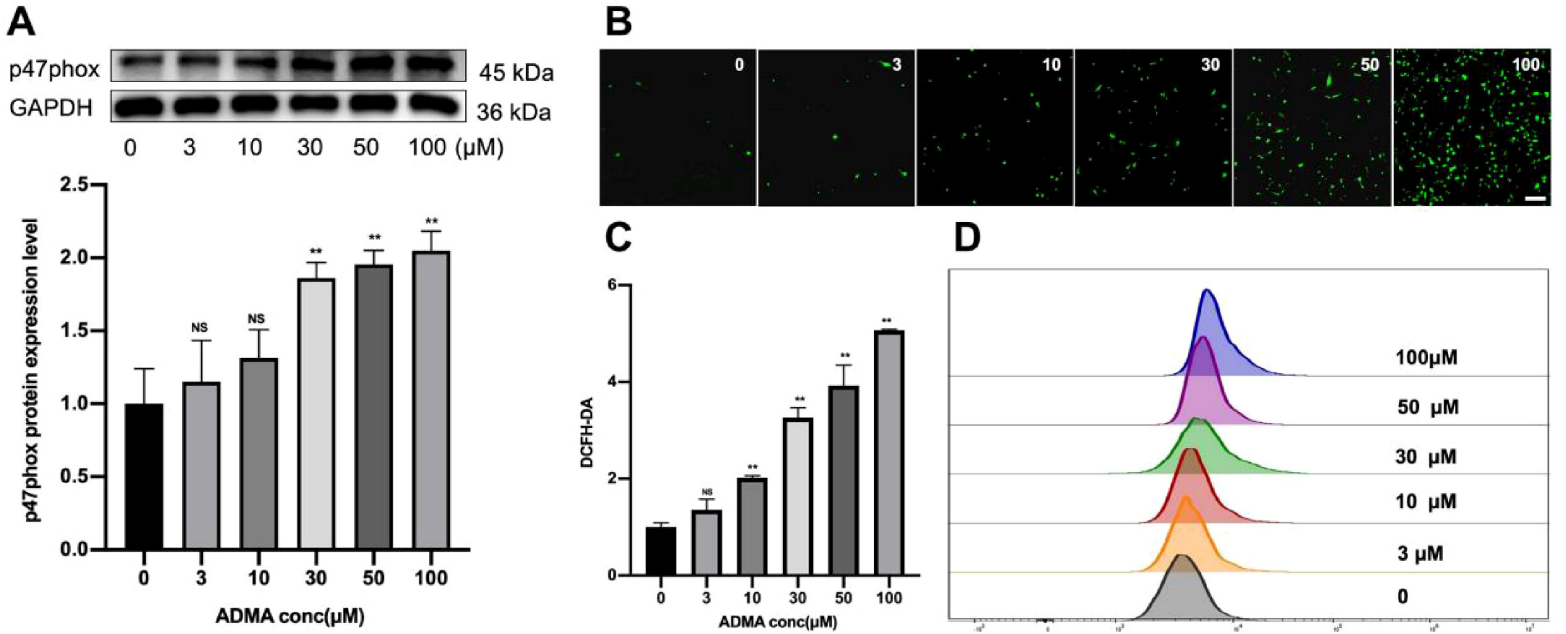
Asymmetric dimethylarginine (ADMA) promotes oxidative stress in HL-1 cells. **(A)** Representative images and a comparison of p47phox protein levels from western blots in six groups. *n* = 5 per group. **(B,C)** The representative images and quantification of ROS in six groups. *n* = 5 per group. scale bar = 50 μm. **(D)** Flow cytometry was utilized to detect ROS levels in HL-1 cells treated with different concentrations of ADMA. Data are presented as mean ± SD. Statistical significance was determined using One-way ANOVA, and Tukey’s post-hoc test. *P < 0.05, **P < 0.01 vs. the normol control group.

Reactive oxygen species, including superoxide anions, hydrogen peroxide, and hydroxyl radicals, are critical byproducts of cellular OS that play a dual role in physiological signaling and pathological damage. In patients with AF, the atrial myocardium exhibits significant oxidative damage, characterized by lipid peroxidation, protein carbonylation, and DNA oxidation, as evidenced by elevated biomarkers in clinical studies (11). To elucidate the molecular mechanisms linking ROS overproduction to AF progression, we quantified key oxidative stress markers-specifically, the NADPH oxidase subunit p47phox and intracellular ROS levels-in HL-1 atrial myocyte cell lines under controlled experimental conditions. ADMA treatment (30–100 μM) significantly induced p47phox expression, a regulatory component of NADPH oxidase, with peak protein levels observed at 100 μM (*P* < 0.01, Figure 2A), suggesting that ADMA activates this major ROS-generating enzyme system. Fluorescence intensity assays using the redox-sensitive dye DCFH-DA revealed a dose-dependent elevation of ROS across treatment groups (*P* < 0.05, Figures 2B, C), with the 100 μM ADMA group showing a 2.1-fold increase compared to controls. Flow cytometry analysis further confirmed maximal ROS production at 100 μM ADMA (Figure 2D), demonstrating that cells exhibited high oxidative activity, which aligned with quantitative microscopic observations of DCF fluorescence. These consistent multi-methodological data demonstrate a clear ADMA concentration-dependent pattern of ROS generation in atrial myocytes, implicating ADMA as a potential mediator of oxidative injury in AF pathogenesis.

### 3.3 ADMA promotes atrial remodeling by facilitating ROS generation

**FIGURE 3:**
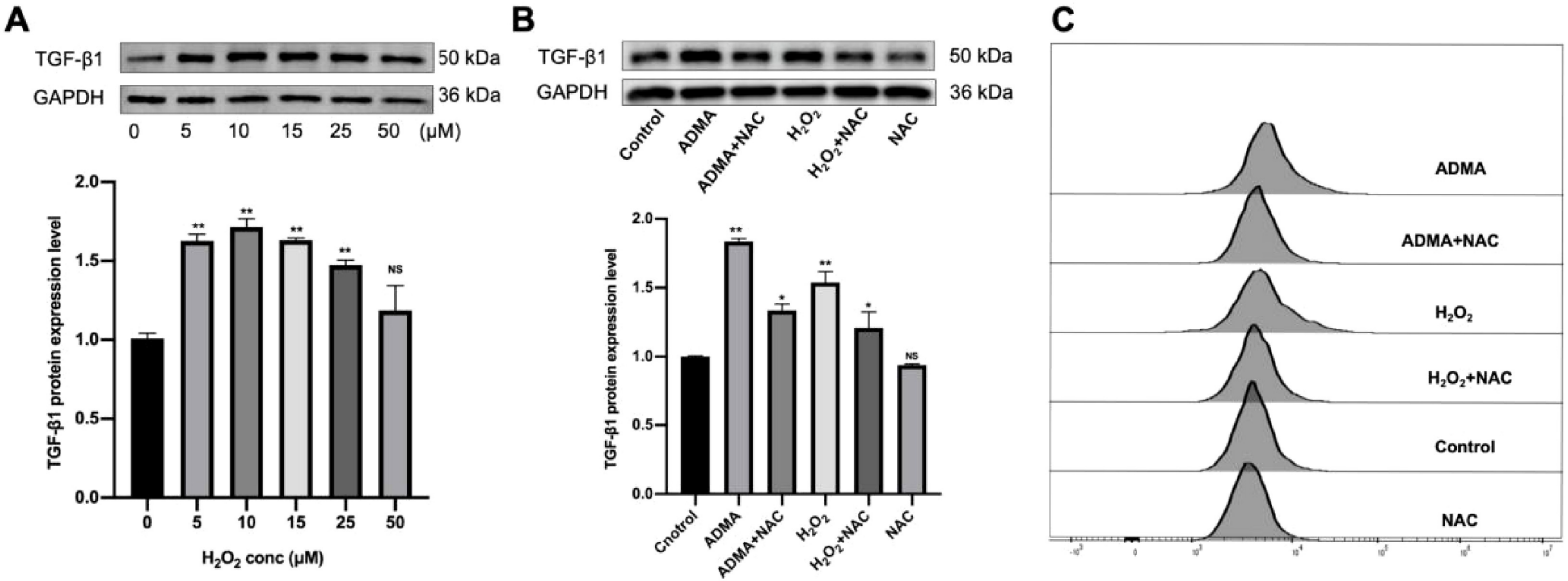
Asymmetric dimethylarginine (ADMA) promotes atrial remodeling by facilitating reactive oxygen species (ROS) generation. **(A)** The level of transforming growth factor-β1 (TGF-β1) was evaluated under varying H_2_O_2_ concentrations (0, 5, 10, 15, 25, 50 μM). *n* = 5 per group. **(B)** The representative images and quantification of TGF-β1 in six groups. *n* = 5 per group. **(C)** Flow cytometry was utilized to detect ROS levels in HL-1 cells. Data are presented as mean ± SD. One-way analysis of variance (ANOVA), and Tukey’s post-hoc test. *P < 0.05, **P < 0.01 vs. the normol control group.

Given that ROS can activate latent TGF-β1 through two distinct mechanisms: the direct oxidation of latency-associated peptides (LAP) and the indirect stimulation of matrix metalloproteinases (MMP-2/9) (12, 13). As a crucial mediator of ROS signaling, hydrogen peroxide (H_2_O_2_) plays a central role in cellular oxidative stress responses. To investigate the regulation of TGF-β1, we treated HL-1 cells with varying concentrations of H_2_O_2_ (0–50 μM), both in the presence and absence of NAC, for 24 h. Initial testing with elevated H_2_O_2_ levels (25–100 μM) induced significant apoptosis (*P* < 0.05) but did not alter TGF-β1 expression. Further dose-response studies revealed that moderate H_2_O_2_ concentrations (5–25 μM) significantly enhanced TGF-β1 production (*P* < 0.05, Figure 3A). To examine NAC’s role in ADMA-induced ROS/TGF-β1 effects, cells were divided into six groups: control, ADMA (30 μM), ADMA + NAC (50 μM), H_2_O_2_ (10 μM), H_2_O_2_ + NAC (50 μM), and NAC (50 μM). After 1-h NAC pre-incubation, ADMA/H_2_O_2_ treatment for 24 h showed that NAC significantly suppressed TGF-β1 upregulation (*P* < 0.05, Figure 3B) and ROS elevation but did not fully reverse them (*P* < 0.05, Figure 3C). NAC alone had no effect on TGF-β1 (*P* < 0.05), confirming ADMA’s ROS-dependent TGF-β1 activation and NAC’s partial blockade. In summary, this study demonstrates that ADMA may activate latent TGF-β1 in atrial myocytes, thereby promoting atrial fibrosis through the enhancement of ROS synthesis.

### 3.4 Atrial fibrillation patients presents with a higher level of serum ADMA and TGF-β1

**FIGURE 4:**
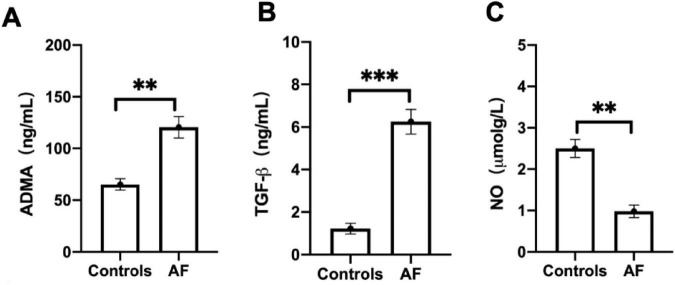
Atrial fibrillation (AF) patients promotes the expression of serum Asymmetric dimethylarginine (ADMA) and transforming growth factor-β1 (TGF-β1). **(A)** The level of ADMA was evaluated between AF patients and controls. **(B)** The level of TGF-β1was evaluated between AF patients and controls. **(C)** The level of nitric oxide (NO) was evaluated between AF patients and controls. Data are presented as mean ± SD. One-way analysis of variance (ANOVA), and Tukey’s post-hoc test. *P < 0.05, **P < 0.01 vs. the normol control group.

The baseline characteristics table (Table 1) reveals significant differences between the 60 patients with AF and the 30 control subjects. Patients with AF exhibited substantially higher rates of hypertension (75% vs.10%), diabetes (31.6% vs.16.7%), and a history of cardiovascular events (*P* < 0.001). Furthermore, the prevalence of medication usage was significantly greater among AF patients, particularly for ACE inhibitors/angiotensin receptor blockers (ACEI/ARB) (66.7% vs. 13.3%) and statins. Laboratory findings indicated markedly elevated triglyceride levels in AF patients (4.25 vs. 2.60 mmol/L, *P* < 0.001). As illustrated in Figure 4, patients with AF exhibit significantly elevated serum concentrations of ADMA (*P* < 0.01, Figure 4A), TGF-β1 (*P* < 0.001, Figure 4B) and decreased NO (*P* < 0.01, Figure 4C) compared to control subjects. The marked increase in these two factors suggests their potential involvement in the pro-oxidant and pro-fibrotic pathways underlying the pathophysiology of AF.

**TABLE 1:** Baseline characteristics of the atrial fibrillation (AF) patients and controls.

Variables	AF patients *N* = 60	Controls *N* = 30	*P*-value
**Baseline characteristics**			
Atrial fibrillation, *n* (%)	60 (66.7%)	30 (33.3%)	–
Age, years	70.25 ± 9.58	68.96 ± 10.37	0.658
Male, *n* (%)	42 (70%)	19 (63.3%)	0.807
Hypertension, *n* (%)	45 (75%)	3 (10%)	< 0.001
Diabetes, *n* (%)	19 (31.6%)	5 (16.7%)	< 0.001
History of old MI, *n* (%)	4 (31.6%)	0	< 0.001
Old stroke, *n* (%)	8 (31.6%)	0	< 0.001
Medication			
AECI or ARB, *n* (%)	40 (66.7%)	4 (13.3%)	< 0.001
Betablocker, *n* (%)	24 (40%)	2 (6.7%)	< 0.001
Statins, *n* (%)	50 (40%)	6 (20%)	< 0.001
**Laboratory examination**			
Creatinine, μmol/L	82.56 ± 5.95	75.25 ± 6.23	0.4
TG, mmol/L	4.25 ± 0.55	2.60 ± 0.30	< 0.001
LDL-C, mmol/L	2.38 ± 0.21	2.02 ± 0.15	0.347

TG, triglyceride; LDL-C, low-density lipoprotein cholesterol.

## 4 Discussion

Atrial structural remodeling, characterized by fibrosis, plays a critical role in the pathogenesis of AF (14, 15). Although initially attributed to the renin-angiotensin-aldosterone system (RAAS), TGF-β1, OS, and matrix metalloproteinases (MMPs), emerging evidence suggests that endothelial dysfunction-marked by reduced NO bioavailability-serves as a pivotal mechanism in this process (16–18). ADMA, an endogenous inhibitor of NOS, decreases NO synthesis and is correlated with the progression of AF. Notably, atrial fibroblasts demonstrate greater sensitivity to TGF-β1 and angiotensin II (AngII) compared to their ventricular counterparts (19, 20). Furthermore, TGF-β1 overexpression selectively induces atrial fibrosis in murine models (21, 22). In a canine model of AF, atrial levels of ADMA were positively correlated with the severity of fibrosis, and ADMA was shown to dose-dependently upregulate TGF-β1 in fibroblasts at concentrations ranging from 0 to 10 μM (23). This study further extended the intervention of ADMA (0–100 μM) in atrial myocytes, revealing a significant increase in TGF-β1 levels, which peaked at 30 μM (*P* < 0.05). Higher concentrations of ADMA did not lead to further increases in TGF-β1 but instead reduced myocyte viability in a concentration-dependent manner, indicating a threshold effect on cellular proliferation. These findings suggest that ADMA likely promotes AF through the induction of atrial fibrosis, which contributes to structural remodeling in the atria and facilitates both the initiation and progression of AF.

Emerging evidence indicates that ADMA exhibits concentration-dependent pro-oxidant effects in vascular endothelial cells, facilitating the production of superoxide anions (24–26). Clinically, plasma ADMA levels in patients with AF show significant positive correlations with left atrial diameter, inflammatory markers, and indices of oxidative stress (27). Utilizing an HL-1 atrial myocyte model, this study revealed that ADMA intervention significantly increased intracellular ROS levels in a concentration-dependent manner, with concurrent validation through flow cytometry and fluorescence microscopy. NADPH oxidase (NOX) remains inactive in resting cells but is activated by AngII, and various growth factors and cytokines, thereby driving superoxide generation (28, 29). Vascular NOX isoforms (1, 2, 4, and 5) are all regulated by AngII, and NOX-dependent ROS has been shown to activate AngII type 1 receptors (AT1R) (29). As a pivotal upstream mediator in the pathogenesis of AF, AngII-induced NOX2 activation generates excessive ROS, accelerating rapid pacing-mediated myocardial degradation and interstitial fibrosis, ultimately leading to atrial structural remodeling (30). Our experiments further demonstrated that increasing concentrations of ADMA elicited a graded upregulation of the NOX regulatory subunit p47phox expression, consistent with the trends observed in ROS generation. This corroborates the role of ADMA in oxidative stress through the activation of the NOX pathway. Finally, The data also reveal significantly higher circulating levels of both ADMA and TGF-β in the AF cohort. This finding supports the central hypothesis of the study, which posits that elevated levels of ADMA contribute to a pro-fibrotic environment in the atria. This contribution may occur through the synergistic action with or upregulation of key mediators such as TGF-β1, thereby accelerating the structural remodeling that perpetuates AF.

Taken together, ADMA promotes both electrical and structural remodeling of the atria through various molecular pathways. This occurs via the inhibition of NOSactivity, leading to a reduction in NO production, which in turn induces oxidative stress and endothelial dysfunction. Consequently, these changes increase susceptibility to atrial fibrillation. The mechanistic network that targets key nodes of the ADMA-NO pathway not only provides novel insights into the pathophysiological mechanisms underlying atrial fibrillation but also presents potential therapeutic targets for the development of innovative prevention and treatment strategies.

## 5 Conclusion

The findings suggest that ADMA likely induces the expression of transforming TGF-β1 through a mechanism that involves the enhancement of NADPH oxidase-derived reactive oxygen species (NOX-ROS) levels. This process, in turn, contributes to myocardial oxidative damage and atrial remodeling.

## 6 Limitation

This study has several limitations: (a) It is lacking validation by animal models; (b) Pathway-specific pharmacological tools were not utilized to verify the underlying mechanisms; (c) The mechanism of action of NAC remains inadequately elucidated; (d) The duration of the ADMA intervention was relatively short, which may not accurately reflect long-term effects. Further validation in animal models is necessary, along with a comprehensive exploration of the interaction mechanisms within the ADMA-ROS-TGF-β1 pathway. Future studies are warranted to address these important issues.

## Data Availability

The datasets used and/or analyzed during the current study available from the corresponding author on reasonable request.
