# Supplementary material for: Asymmetric dimethylarginine mediates oxidative stress and atrial remodeling in HL-1 cells
Source: Front Med (Lausanne). 2025 Nov 4;12:1696845. doi: 10.3389/fmed.2025.1696845 (PMC12631613; doi:10.3389/fmed.2025.1696845)

1. The research used 3-colorpre-dyed protein Marker (PR1960)

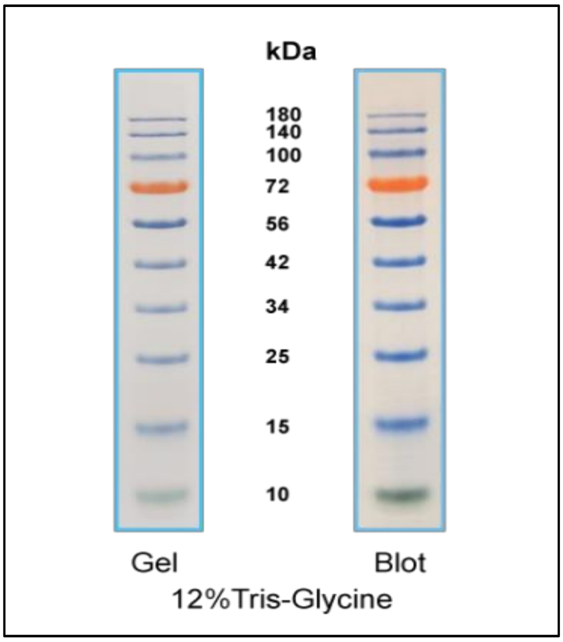

2.Western gels/blots are used in Figure 1 B.

Cytoplasm TGF-β1(50kDa)

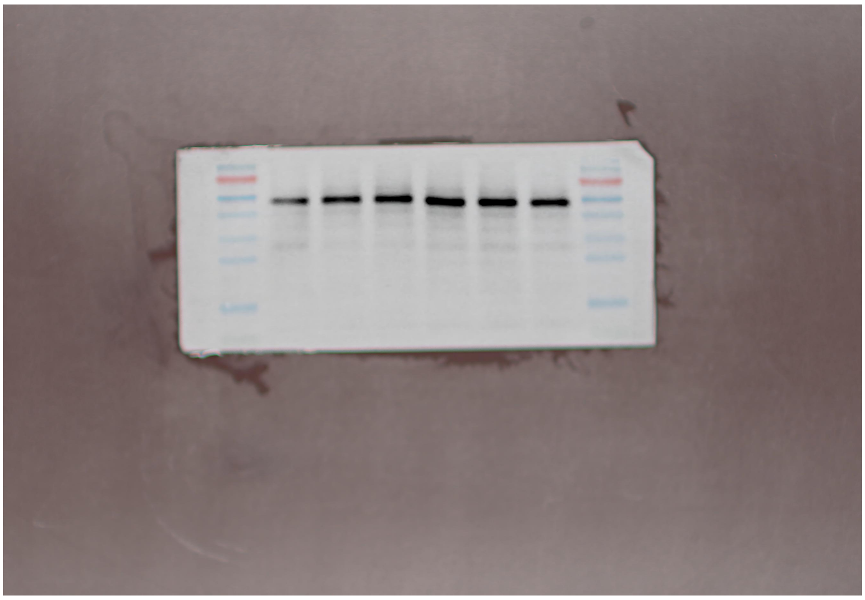

GAPDH (36kDa)

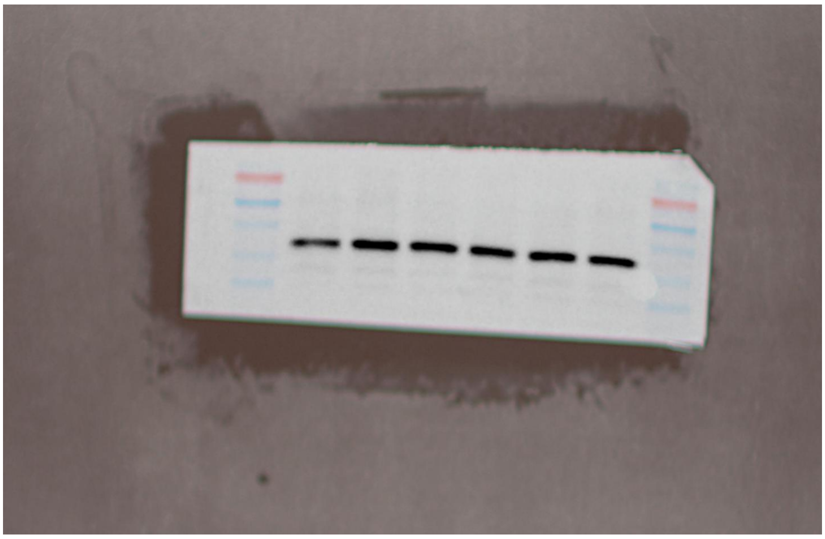

3.Western gels/blots are used in Figure 2 A .

p47phox (45kDa)

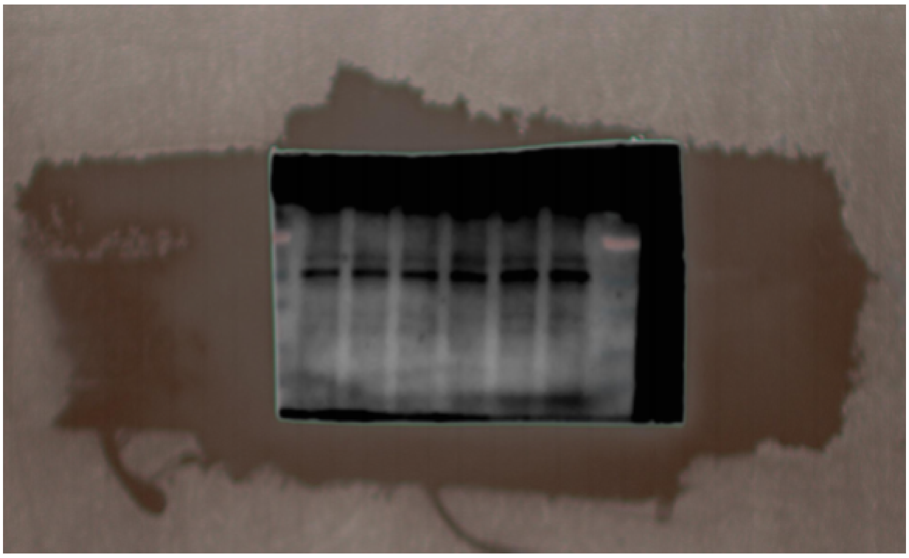

GAPDH (36kDa)

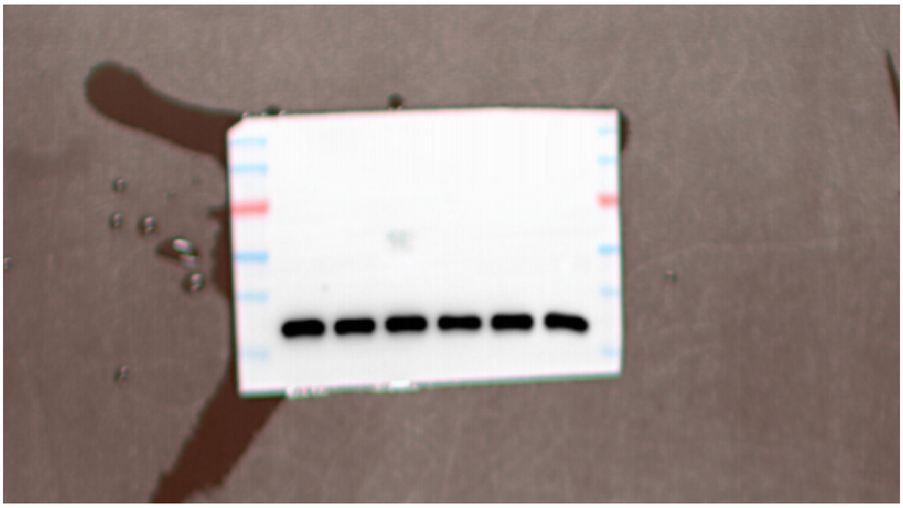

4.Western gels/blots are used in Figure 3 A .

Cytoplasm TGF-β1(50kDa)

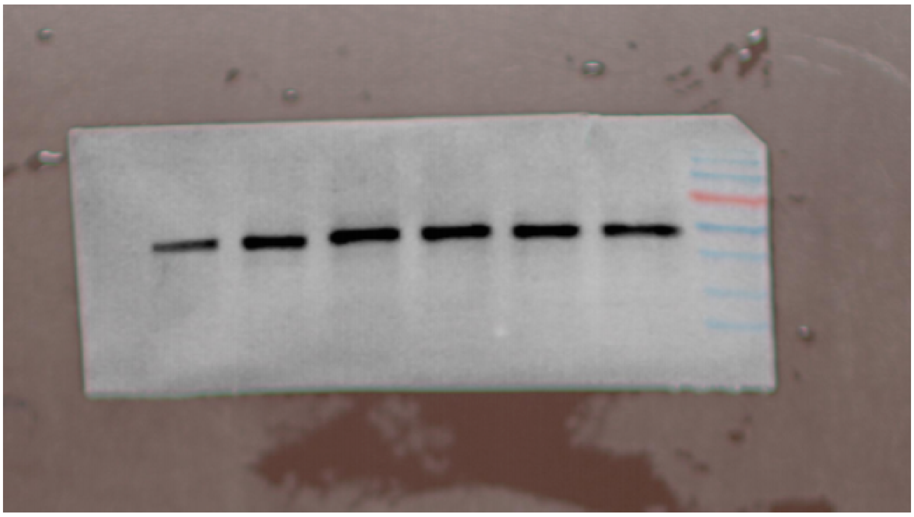

GAPDH （36kDa)

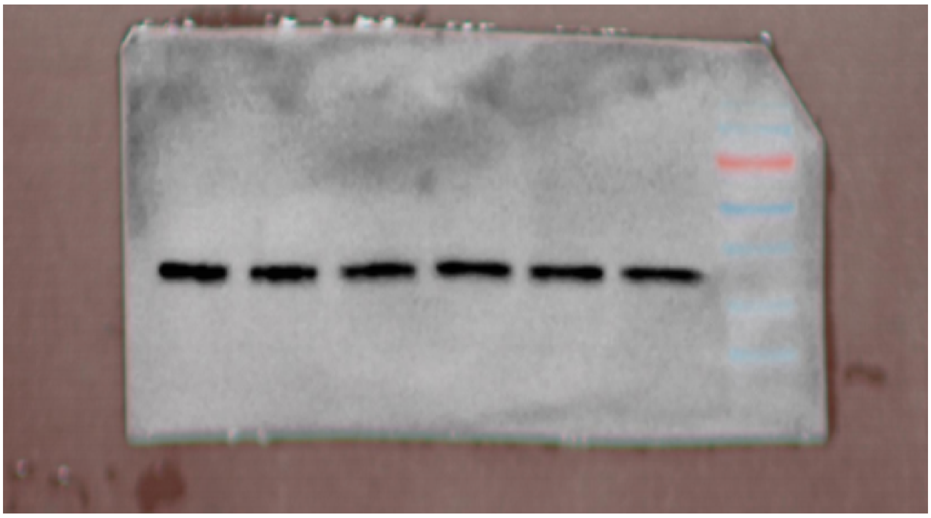

5.Western gels/blots are used in Figure 3 B.

Cytoplasm TGF-β1(50kDa)

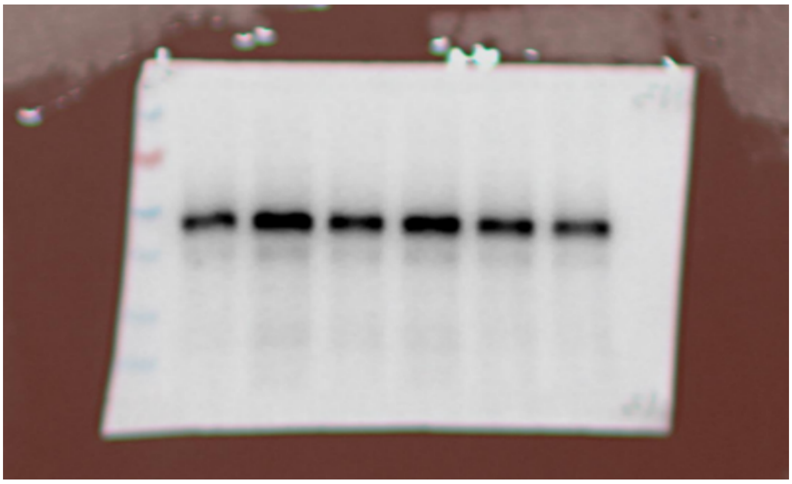

GAPDH （36kDa)

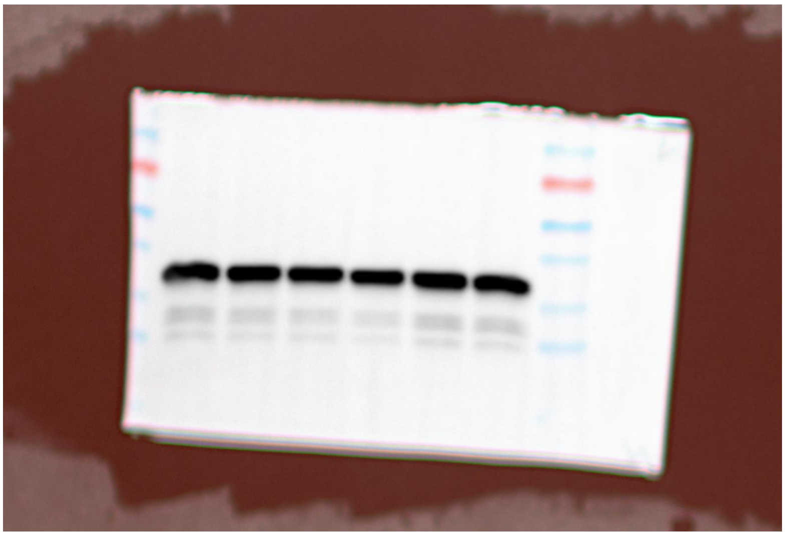

Supplement: Supplementary file 1 [file Data_Sheet_1.pdf]
